# Case Report: Novel splicing mutations in *RFX5* causing MHC class II deficiency

**DOI:** 10.3389/fgene.2022.978688

**Published:** 2022-10-07

**Authors:** Shan Chen, Yuqing Xu, Yeqing Qian, Zhaohui Li, Minyue Dong

**Affiliations:** ^1^ Laboratory of Prenatal Diagnosis, Mindong Hospital Affiliated to Fujian Medical University, Ningde, Fujian, China; ^2^ Women’s Hospital, School of Medicine, Zhejiang University, Hangzhou, China; ^3^ Key Laboratory of Reproductive Genetics, Ministry of Education (Zhejiang University), Hangzhou, China

**Keywords:** RFX5, MHC-II deficiency, splicing mutation, DNA-binding domain, whole exome sequencing

## Abstract

Mutations of the Regulatory Factor X5 (*RFX5*) have been associated with the autosomal recessive major histocompatibility class II (MHC-II) deficiency, which is a severe immunodeficiency characterized by constitutive and interferon-gamma induced MHC II expression disorder and leads to the absence of cellular and humoral T-cell response to antigen challenge. The compound heterozygous splicing mutations of *RFX5*: c.353 + 6T>G (maternally inherited) and c.757 + 1G>A (paternally inherited) were identified in an infant diagnosed severe immunodeficiency. The mutation c.757 + 1G>A was classified as likely pathogenic while c.353 + 6T>G was classified as the variant of uncertain significance according to American College of Medical Genetics and Genomics (ACMG). To investigate the pathogenicity of *RFX5*: c.353 + 6T>G, reverse transcription PCR (RT-PCR) was conducted with the mother’s peripheral blood. An insertion of 191-bp intronic sequence (intron 6) was found in the transcripts, and this resulted in a frameshift and premature truncation of the protein, especially reduced the DNA-binding domain (DBD) of the RFX5 protein. Our data expanded the spectrum of pathogenic mutations in MHC-II deficiency and put new insights into the genetic counseling, prenatal diagnosis and preimplantation genetic testing (PGT) for the disease.

## Introduction

Regulatory factor X-5 (*RFX5*) is essential for the regulation of major histocompatibility class II (MHC II) gene expression (Villard et al., 1997; Djidjik et al., 2012). RFX5 contains highly conserved DNA-binding domains (*DBDs*), located in the 90–166 residues and 407–614 residues, which bind the X box of MHC II before transcription (Clarridge et al., 2016; Farrokhi et al., 2018).

Mutations in *RFX5* have been associated with the MHC-II deficiency, also named as the Bare Lymphocyte Syndrome (BLS) (OMIM:209,920) (Reith and Mach, 2001; Nekrep et al., 2002). MHC-II deficiency, a rare autosomal recessive disease, is characterized by constitutive and interferon-gamma induced MHC II expression disorder, and results in the absence of cellular and humoral T-cell response to antigen challenge, hypogammaglobulinemia and impaired antibody production (Garvie et al., 2007; Hanna and Etzioni, 2014). Over 200 cases have been reported and the prevalence varies significantly in different regions based on previous published data. Around two-thirds of the patients come from North Africa while less than 10 cases have been reported in East Asia (Djidjik et al., 2012; El Hawary et al., 2019). Children with MHC-II deficiency are extremely susceptible to a broad range of viral, bacterial and fungal, among which *Pneumocystis jirovecii, Salmonella*, cytomegalovirus *(CMV), Cryptosporidium* species and *herpes simplex virus* (HPV) are the most common pathogens (Hanna and Etzioni, 2014). Therefore, these patients are mainly characterized by severe and recurrent infections within the first year of life, especially involving the respiratory and gastrointestinal tract. What’s worse, the infection may be lethal (Reith and Mach, 2001; Farrokhi et al., 2018).

In the current investigation, we described a Chinese infant with MHC II deficiency caused by two novel splicing mutations, c.353 + 6T>G (maternally inherited) and c.757 + 1G>A (paternally inherited) in the *RFX5* gene.

## Methods

### Subject

A 30-year-old healthy woman who delivered an infant (the proband) diagnosed severe immunodeficiency was referred to the Department of Reproductive Genetics, Women’s Hospital, School of Medicine Zhejiang University. Her infant presented recurrent pneumonia, reduced CD3 and CD4 positive leucocyte cell ratio, inverted CD4/CD8 ratio and reduced serum immunoglobulins levels (concentrations of IgG, IgA, and IgM) at 6 months of her age (Table 1). The infant died at 22 months of her age due to severe respiratory infection and respiratory failure. Severe immunodeficiency was diagnosed with unknown cause. The infant was born at full term to healthy un-consanguineous Chinese parents without family history of any genetic disorders.

**TABLE 1:** Abnormal immunological findings of the infant with the associated normal range.

	Patient data	Normal range	Unit
Cell count			
White Blood Cell	17.81	4–10	10^9^/L
Lymphocyte	2.91	0.8–4	10^9^/L
Monocyte	0.74	0.12–1.2	10^9^/L
Platelet	351	100–300	10^9^/L
Immunophenotyping			
CD3	38	55–84	%
CD4	11	31–60	%
CD8	26	13–41	%
CD4/CD8 ratio	0.44	0.8–2.8	
Ig concentration			
IgG	0.41	5.2–16	g/L
IgA	0.02	0.24–3.3	g/L
IgM	0.22	0.5–2.2	g/L

The use of medical records of this family is was approved by the Institutional Review Board of the Women’s Hospital, School of Medicine, Zhejiang University and the participants provided their written informed consents.

### WES and bioinformatic analysis

To determine the cause for severe immunodeficiency, the whole exome sequencing (WES) was provided. Genomic DNA from all the family members was extracted by a QIAamp DNA blood midi kit (Qiagen, Hilden, Germany) according to the manufacturer’s protocol and then was fragmented by Covaris LE220 (Massachusetts, United States) to generate a paired-end library (200–250 bp). All amplified libraries were performed on the BGISEQ-500 platform (BGI, Shenzhen, China), the single-strand DNA was mixed with MGIEasy™ DNA Library Prep Kit V1 (BGI, Shenzhen, China) and then sequenced using 100SR chemistry with BGISEQ-500RS high-throughput sequencing Kit (BGI, Shenzhen, China).

Splice AI (https://spliceailookup.broadinstitute.org/) was used to predict the effect of variants. Pathogenic variants are assessed according to the protocol issued by the American College of Medical Genetics and Genomics (ACMG) (Richards et al., 2015). DECIPHER (http://decipher.sanger.ac.uk), OMIM (http://omim.org/), PubMed (http://www.ncbi.nlm.nih.gov/pubmed), ClinVar (https://www.ncbi.nlm.nih.gov/clinvar/), and HGMD (http://www. hgmd. cf.ac.uk/ac/index.php) databases were used to investigate the clinical relevance of the mutations.

### Sanger sequencing validation

Sanger sequencing was carried out to confirm the variants in *RFX5* gene. The primers used for c.757 + 1G>A were as follows: *RFX5*-E9F, TAG​CTG​AGG​CAG​AGG​ATG​AAG​A; and *RFX5*-E9R, GGT​GAG​GAG​GAA​ACT​GAG​GAA​T. The primers used for c.353 + 6T>G were as follows: *RFX5*-E5F, GTT​AGG​GTC​TTA​GTA​ATG​CTT​GTT​CC; and *RFX5*-E5R, CCT​TCG​AGC​TTT​GAT​GTC​AGG. The primers were designed using Oligo Primer Designer (Rychlik 2007). The DNA was amplified using the following procedure: 94°C for 10 min; 35 cycles at 94°C for 30 s, 60°C for 30 s, 72°C for 30 s; 72°C for 10 min. Sequencing was performed by an ABI 3130 DNA analyzer.

### RNA extraction, real time-PCR, and sequencing

Total RNAs of the mother’s peripheral blood cells was extracted using TRIzol (Takara, Japan). Extracted total RNAs was reverse-transcribed using RT Kit (Takara, Japan) following the manufacturer’s instructions. RT-PCR was performed using GoldStar Best Master Mix (CWBIO, Beijing). Sequences of primers used were as follows: RFX5-spF, GAA​GAT​GAG​CCT​GAT​GCT​AAG​AG; and RFX5-spR, GGC​GAC​CTC​AAC​GAT​GGA​AC. The procedure of the PCR was as follows: 94°C for 10 min followed by 35 cycles at 94°C for 30 s, 60°C for 30 s, 72°C for 30 s, and a final extension step at 72°C for 10 min. Sequencing was performed by an ABI 3130 DNA analyzer.

## Results

### Identification of the compound heterozygous mutations in *RFX5*

**FIGURE 1:**
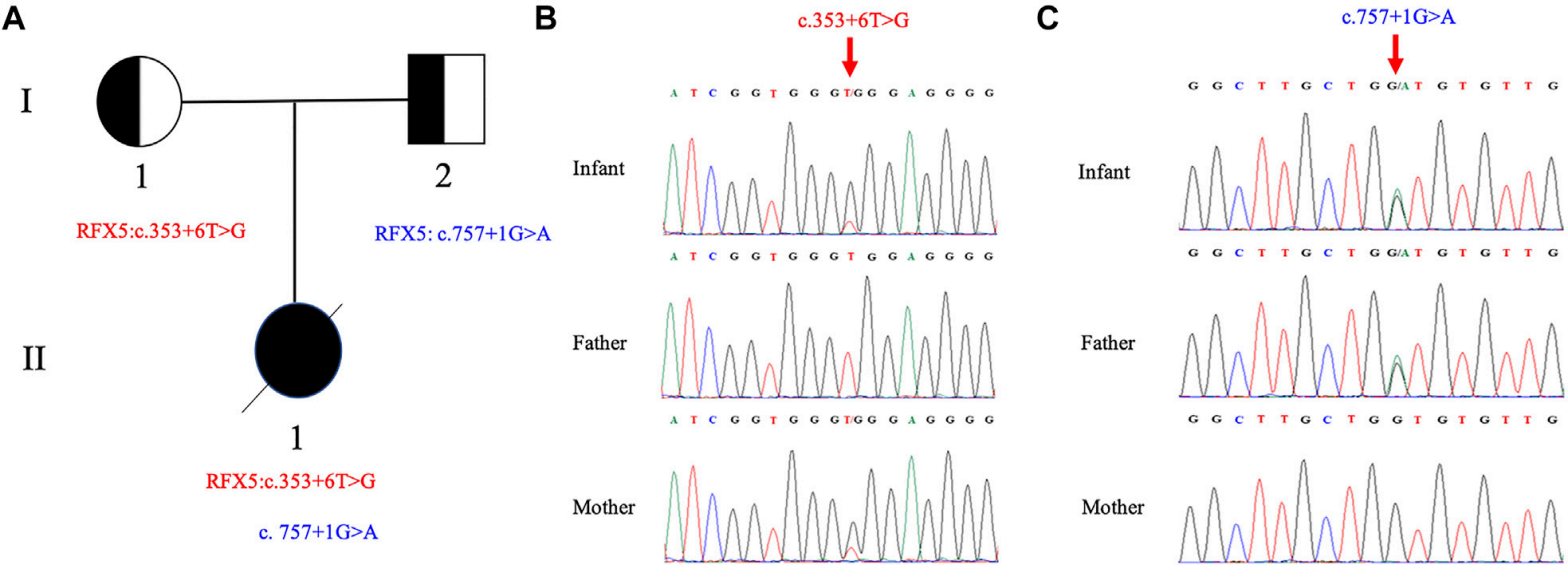
Pedigree of the family and Sanger sequence analysis. **(A)** II_1_(proband) have two compound splicing variants in *RFX5* gene. One variant (c.353+6T > G) is from I_1_ (mother) and the other (c.757+1G > A) is from I_2_ (father). Chromatograms of **(B)** c.353+6T > G and **(C)** c.757+1G > A are identified by Sanger sequencing, respectively. The top chromatograms are from the infant, the middle two are from the father, and the bottom two are from the mother (red arrows indicate the mutation).

Compound heterozygous splicing mutations in *RFX5*: c.353 + 6 T>G and c.757 + 1 G>A were identified by WES and confirmed by Sanger sequencing (Figure 1). These mutations have never been reported in any database (gnomAD, ClinVar or HGMD) or literature. Splice AI was used to predict the effects of the *RFX5*: c.353 + 6T>G and c.757 + 1G>A on splicing. The delta score of donor loss were 0.79 and 0.85, respectively. The result indicates that both of the mutations affect the splicing. The mutation *RFX5*: c.757 + 1G>A was inherited from her father and classified as likely pathogenic, while the mutation *RFX5*: c.353 + 6T>G was maternally inherited and classified as variant of uncertain significance (VUS) according to ACMG recommendations.

### Pathogenicity of *RFX5*: c.353 + 6T>G

**FIGURE 2:**
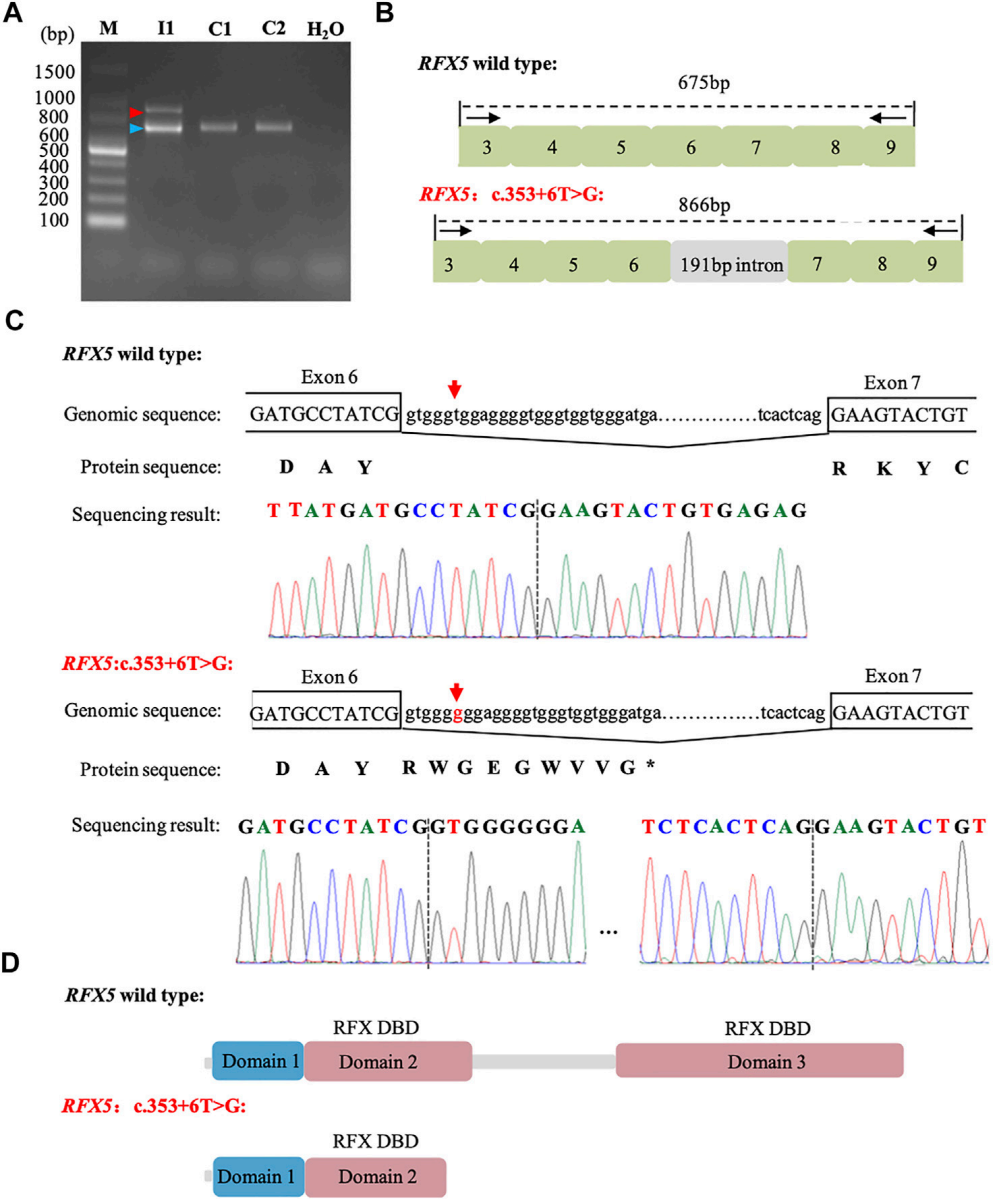
Analysis of c. 353 + 6T>G splicing mutation in *RFX5*. **(A)**: RT-PCR analysis of *RFX5* cDNA from peripheral blood samples. Agarose gel (1.5%) electrophoresis of RT-PCR products generated from I_1_ (mother), C1 and C2 (normal controls). Amplicons resulting from aberrantly spliced mRNA and normal spliced mRNA are marked by red and blue arrowheads, respectively. **(B)**: Schematic representation of exon three to nine and intron six organization in RFX5. **(C)**: Sequence analysis of the RT-PCR products from the mother. The arrows indicate the position of the c. 353 + 6T>G mutation. **(D)**: The structure with domains of wild-type and c. 353 + 6T>G splicing mutation RFX5 protein. Wild-type RFX5 protein consists of three domains, among which domain two and domain three are the important DBDs while the splicing mutation leads to the premature of the protein and significantly damages the protein structure.

Based on the genotype–phenotype correlation, we hypothesized that *RFX5*: c.353 + 6T>G may affect the splicing. To confirm this hypothesis, mRNA was extracted from the women’s peripheral blood cells. RT-PCR was performed with the primers (RFX5-spF/RFX5-spR) designed to amplify exons three to nine of *RFX5*. It was found that the woman (I_1_) and the controls (C1 and C2) shared the band of PCR products at 675 bp, while I_1_ had another bigger band of 866 bp (Figure 2A). The Sanger sequencing of the bigger band (866 bp) showed that 191-bp intron six sequences were retained from the transcripts of the mother, compared with the 675 bp band (Figures 2B,C). The mutation (c.353 + 6T>G) introduced an insertion of 191-bp intron six sequences, which may cause a truncated RFX5 protein by a frameshift and creation of a premature stop codon. As is showed in Figure 2D, wild-type deduced RFX5 protein has three domains, among which two domains are DNA-binding domains (DBDs). However, the deduced RFX5 protein of c.353 + 6T>G splicing mutation only has one truncated DBD. The truncated DBD may damage the ability of RFX5 to bind X box of the MHC II promoter and then reduce the expression of MHC II molecular at the transcriptional level.

## Discussion

In the current investigation, we described a Chinese infant with MHC class II deficiency due to the compound heterozygous splicing mutations of the *RFX5* gene for the first time. The paternally inherited mutation c.757 + 1G>A of *RFX5* was likely pathogenic and the maternally inherited mutation c.353 + 6T>G was proved to affect splicing, which may result in frameshift and truncation of the protein. Both of the mutations have never been reported in any database or literature, indicating our findings expand the spectrum of the diagnose for the MHC II deficiency and provide insight and information for genetic counseling.

Major histocompatibility complex (MHC) II deficiency is a primary immunodeficiency with an autosomal recessive inheritance pattern and is characterized by the early onset of severe and recurrent respiratory and gastrointestinal infections, developmental delay, and death in early life (Plaeger-Marshall et al., 1988; Lum et al., 2020). Almost all of the patients suffer from recurrent pneumonia and prolonged diarrhea (Clement, 1990; Ben-Mustapha et al., 2013). In the present study, the infant presented typically clinical and immunological features, like severe pneumonia, reduced CD3 and CD4 positive leucocyte cell ratio, inverse CD4/CD8 ratio and reduced serum immunoglobulin levels (concentrations of IgG, IgA, and IgM). The infant eventually died at 22 months of age due to acute respiratory infection and respiratory failure.

The underlying cause of MHC class II deficiency lies in the absence or reduced expression of MHC class II molecules which are regulated by MHC II enhanceosome (a cell-specific multiprotein complex) (Masternak et al., 2000; Hanna and Etzioni, 2014). The MHC class II molecules, also referred to as human leukocyte antigens (HLAs), are multigenic and highly polymorphic glycoproteins that aggregate to form heterodimers of *a* and β chains (Hanna and Etzioni, 2014). Moreover, HLAs are usually divided into three molecules, HLA-DR, -DP, and -DQ, which are located on the surface of antigen-presenting cells (APCs) such as dendritic cells and macrophages (Waldburger et al., 2000). HLAs present antigens endocytosed by APCs to the receptor of CD4^+^ helper T cells, directing the T cell activation, differentiation, and proliferation (Villard et al., 2000). It is reported that MHC class II gene mutations might damage cellular and humoral immunity by affecting CD4 T-cell development and reducing the Th-cell-dependent antibody production (Nonoyama et al., 1998; Sage et al., 2014; Aluri et al., 2018). Class II transactivator (*CIITA*), RFX-associated protein (*RFXAP)*, regulatory factor X-5 (*RFX5*), and RFXAP-containing ankyrin repeat (*RFXANK*) are widely recognized key enhanceosome of MHC class II molecules so far. Accordingly, based on the four different transcript factors, MHC II deficiency is divided into four groups from group A to D, which are summarized by the deficiency of *CIITA, RFXANK, RFX5*, and *RFXAP*, respectively (Steimle et al., 1995; Scholl et al., 1997). In our study, two splicing mutations (c. 757 + 1G>A and c. 353 + 6T>G) in *RFX5* gene were found in the proband, who belongs to group C. The former mutation was located at the classical splicing site, and the latter mutation was proven to lead to a stop codon after amino acid 126, leading to a loss of more than 50% of the protein including the highly conserved DBD. Therefore, the ability of *RFX5* to bind X box could be affected and the expression of MHC II molecular reduced. Taken together, the compound heterozygous mutations in proband might explain the cause for immune deficiency.

Up to now, 19 pathogenic/uncertain significance mutations have been reported in RFX5(HGMD Professional 2022.2). Among them, are five missense mutations (two pathogenic mutations and three uncertain significance mutations), four nonsense mutations (all pathogenic mutations), five splicing mutations (all pathogenic mutations), four small deletions mutations (three pathogenic mutations and one uncertain significance mutation) and one small insertions mutation (pathogenic mutation). The five splicing mutations are c.116 + 1G>A, c.151-1G>A, c.234-1G>A, c.556–2A>G and c.116 + 5G>A, respectively. Four of them are of the classical splice site variants, the last one (c.116 + 5 G>A) is a point mutation in a splice donor site, which results in 10 nucleotide upstream in exon two deletion in *RFX5* mRNA (Villard et al., 1997). The splicing mutations reported in this study are novel.

Hematopoietic stem cell transplantation (HSCT) is currently the only available curative treatment for MHC-II deficiency. However, the success rate is reported to be poor in MHC class II-deficient patients (Small et al., 2013; Posovszky et al., 2019). On the basis of a previous study, two patients did not survive although they underwent HSCT after diagnosis. One patient died of diarrhea and Gram-negative sepsis within 8 days of transplant procedure and the other died post-HSCT due to lung damage and systemic candidiasis (Aluri et al., 2018). Even though the patients with MHC II deficiency do not express MHC II required for the rejection process, the residual host immunity is sufficient to cause rejection even with immunosuppression. In a recent study, it was suggested that the low survival rate in these patients may lie in the presentation of donor antigens by donor antigen-presenting cells to recipient Th cells leading to graft rejection. (Kallen and Pullarkat, 2015). Apart from poor engraftment, a high rate of post-HSCT death can be caused by diagnosis and/or treatment delays, multiorgan failure and persistent viral infections. More importantly, pregnant women with immunodeficiency fetuses mostly experience normal prenatal examination in imaging (ultrasound or MRI) and laboratory tests, like the mother in our study. Therefore, it is of great value to carry out prenatal diagnosis or PGD in such families.

In summary, we reported two novel splicing mutations (c.353 + 6T>G and c.757 + 1G>A) in *RFX5* which are associated with MHC class II deficiency. The mutations were predicted to affect the RFX5 protein translation and even result in the premature of the protein. In addition, our study validates that the RT-PCR is necessary if the genotype–phenotype correlation was very consistent while only one classical splicing site gene mutation of autosomal recessive disease was detected. It contributed to a new genetic foundation for prenatal diagnosis and prenatal diagnosis of MHC class II deficiency.

## Data Availability

The datasets for this article are not publicly available due to concerns regarding participant/patient anonymity. Requests to access the datasets should be directed to the corresponding authors.
